# Validation of a novel 3D printed positioning device and dynamic radiographic technique to quantify rotational laxity of the stifle in dogs

**DOI:** 10.3389/fvets.2023.1118755

**Published:** 2023-03-03

**Authors:** Jin Yu, Dominique J. Griffon, Gary Wisser, Ayman A. Mostafa, Fanglong Dong

**Affiliations:** ^1^College of Veterinary Medicine, Western University of Health Sciences, Pomona, CA, United States; ^2^Center for Excellence in Teaching and Learning, Western University of Health Sciences, Pomona, CA, United States; ^3^Department of Small Animal Surgery and Radiology, Faculty of Veterinary Medicine, Cairo University, Giza, Egypt; ^4^Graduate College of Biomedical Sciences, Western University of Health Sciences, Pomona, CA, United States

**Keywords:** canine, cranial cruciate ligament deficiency, dynamic radiography, tibial torsion, rotation, joint instability, joint laxity

## Abstract

Cranial cruciate ligament deficiency (CCLD) results in internal rotational instability of the stifle (RLS). By contrast, tibial torsion (TT) is an anatomical feature of the tibia along its longitudinal axis. The objective of this study was to validate a dynamic radiographic technique to measure internal rotational laxity of the stifle and differentiate it from TT. Models included transection of the CCL for RLS and an osteotomy for TT. One limb within eight pairs of canine cadaveric hind limbs was randomly assigned to CCLD. The contralateral limb underwent TT, followed by CCLD. Neutral and stress radiographs were taken with the limb in a custom rotating 3-D printed positioning device before and after each modification. The position of the calcaneus on neutral views and the magnitude of its displacement under standardized torque were compared within limbs and between groups. Transection of the CCL increased the magnitude of displacement of the calcaneus by 1.6 mm (0.3–3.1 mm, *p* < 0.05) within limbs. The lateral calcaneal displacement (dS-dN) tended to be greater when CCLD limbs were compared to limbs with intact CCL. A magnitude of calcaneal displacement of 3.45 mm differentiated limbs with RLS from intact limbs with 87.5% sensitivity and 68.7% specificity. The calcaneus was displaced further laterally by about 3 mm on neutral radiographs (dN) when limbs with experimental TT were compared to those without TT (*p* < 0.05). A calcaneus located at least 3.25 mm from the sulcus differentiated limbs with TT from intact limbs with 87.5% sensitivity and 87.5% specificity. The technique reported here allowed detection of RLS, especially within limbs. A calcaneus located at least 3.25 mm on neutral radiographs of large dogs should prompt a presumptive diagnosis of TT.

## Introduction

Cranial cruciate ligament deficiency (CCLD) is the most common cause of lameness and degenerative joint disease in the stifle of dogs with a prevalence that has doubled over the last 30 years (1). The CCL originates from the caudomedial aspect of the lateral femoral condyle and attaches on the mediocranial aspect of the tibia (2). CCLD therefore results in cranial translation of the tibia and internal rotational laxity of the stifle (RLS) with cranial tibial thrust and rotational instability (RIS) during weight bearing (3). The prevalence and morbidity of CCLD seem greater in large-breed than in small breed dogs (1, 4). For instance, Labrador Retrievers have been found predisposed to CCLD, with contralateral disease occurring within 5.5 months in approximately half (48%) of these dogs (4–6). Tibial osteotomies have gained popularity as surgical treatments of CCLD in large dogs, modifying the joint geometry to neutralize cranial thrust of the tibia during weight-bearing (3). However, the influence of these procedures on RIS varies between studies and remains unclear (7–10). A recent study of sixteen dogs treated for CCLD found evidence of persistent cranial tibial thrust in one third (5/16) of the limbs after tibial plateau leveling osteotomy (TPLO), with associated RIS during mid- to late-stance phases (9). This residual rotational instability may contribute to postoperative complications, including postliminary meniscal disease, osteoarthritis, and “pivot-shift” phenomenon (8, 11). The latter is defined as a sudden lateral displacement of the stifle when the dog starts bearing weight on the limb, resulting from a cranial subluxation and tibial internal rotation of the tibia, with lateralization of the hock (12).

Joint laxity is generally defined as increased mobility of the joints to an externally applied force. Laxity tests evaluate the passive envelope of motion of a joint in an unloaded limb without considering muscles and loading activity. On the other hand, joint instability is a kinematic abnormality, occurring with dynamic loading (e.g., weight-bearing) during daily physiological activities (13). These two terminologies are often misused in veterinary medicine. In this article, we use the two terminologies accordingly within the context.

By contrast to the dynamic nature of RIS, tibial torsion (TT) is an anatomical feature, quantifying the twisting of the tibia in relation to its longitudinal axis. The lateral displacement of the calcaneus relative to the sulcus of the talus has been proposed as a radiographic indicator of TT in dogs (14). The presence of TT in dogs with CCLD could justify concurrent correction of it during tibial osteotomy. However, the validity of this approach was subsequently questioned in an *ex-vivo* study of normal bones (15). In this study, Apelt et al. (15) took caudocranial radiographs of normal cadaver limbs after transection of the CCL, effectively creating rotational laxity of the stifle (RLS). Whereas, the medial edge of the calcaneus was aligned with the sulcus of the talus on neutral radiographs, the calcaneus was displaced by approximately 7 mm when the tibia was rotated by 15°. The authors concluded that this measurement should not be used as the sole arbiter of tibial torsion prior to surgical correction because RLS induced similar changes as an experimental tibial osteotomy rotating the tibia by 15° (15). Instead, preoperative computed tomographic examination was recommended to assess the conformation of the tibia before correction of TT. Whereas, cranial laxity of the stifle is routinely assessed in the clinical setting by tibial compression and drawer signs, no cost-effective and simple method has been established to quantify RLS of the canine stifle. This gap in knowledge affects the consistent screening of CCL-deficient dogs for TT, prevents evidence-based management of RLS, and affects the outcomes of dogs treated for CCLD.

The objective of this project is to develop and validate a radiographic technique to quantify RLS and differentiate RLS from TT. We hypothesized that the radiographic position of the calcaneus would be located further laterally relative to the sulcus of the talus when the tibia is maintained in a neutral position (dN) in limbs with tibial torsion compared to intact and CCLD limbs of large dogs. We also hypothesized that the calcaneal displacement would be greater in limbs with RLS than in intact limbs or those with induced tibial torsion when standardized torque is applied to the tibia (dS–dN).

## Materials and equipment

### Positioning device

**Figure 1:**
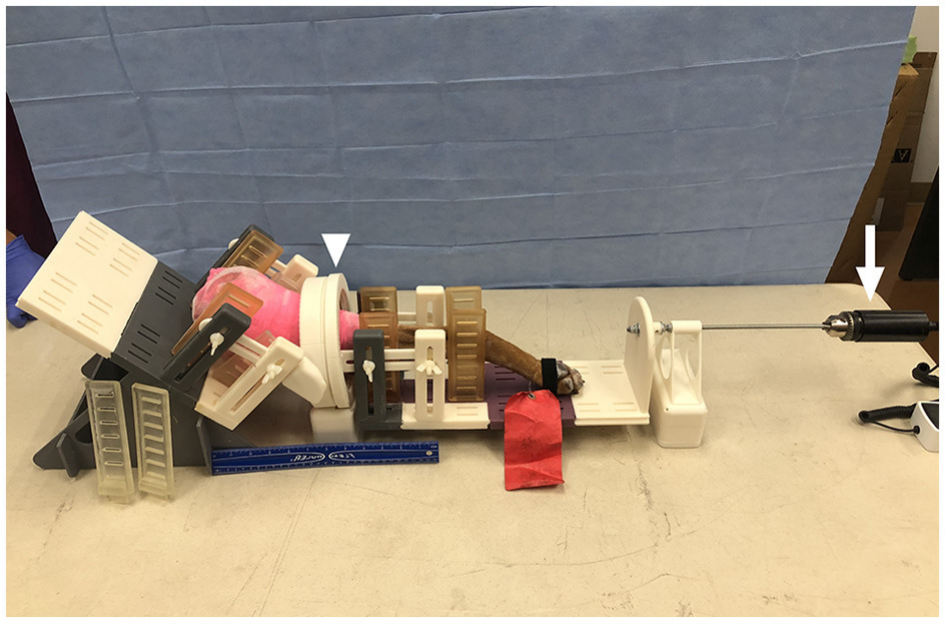
Photograph of a limb in the 3D-printed positioning device with a graduated ring (arrowhead) over the stifle and a carbon rod (arrow) connected to a digital torque. The thigh and crus are immobilized with adjustable rails and tape. Torque is transferred to the tibia through the rails. No implant is placed in the limbs, to allow future clinical application. The stifle did not touch the bearing and the tibia was parallel to the table.

The device was 3D printed with a combination of Formlabs Tough Resin and Color Resin and 12 mm diameter plastic bearings purchased online (Uxcell, Hong Kong, China; Formlabs Inc. Somerville, MA, USA). The device includes medial and lateral adjustable rails and clips, a graduated ring over the stifle and a carbon rod connected to a digital torque meter (model MTT03-12, ABQ Industrial, The Woodlands, TX, USA) (Figure 1). This radiolucent device was designed to maintain the position of the thigh while applying 65 N.cm of predetermined torque on the tibia and to measure the degree of rotation induced at the level of the stifle with the graduated ring. This torque is consistent with the force applied by orthopedic surgeons tightening bone screws (16). Computed tomographic images obtained from a previous project on Labrador Retrievers were reconstructed and served as a model for the device (17). Several iterations of the prototype were tested on cadaver limbs to palliate the following issues: 1interference between the limb support and the radiographic table, 2- friction within the rotating arcs, 3- movement of the limb within the device, and 4-misalignment between the axis of rotation of the device and the tibial axis. The final prototype (Figure 1) was used throughout the study.

### Specimens

All procedures were approved by the Institutional Animal Care and Use Committee (IACUC) of Western University of Health Sciences. Sixteen normal pelvic limbs were obtained from eight large mixed-breed dogs who died of reasons unrelated to the study (Skulls unlimited Inc., Oklahoma City, OK, USA). Pelvic limbs were included if their femurs measured at least 18 cm in length and if no abnormality of the stifle was identified on physical examination and radiographs (17). Medio-lateral views of the femur and tibia, craniocaudal view of the femur and caudocranial view of the tibia were obtained on intact limbs. Radiographic measurements included the lengths of the femur and tibia and the craniocaudal width of the tibial mid-diaphysis.

## Methods

### Study design

**Figure 2:**
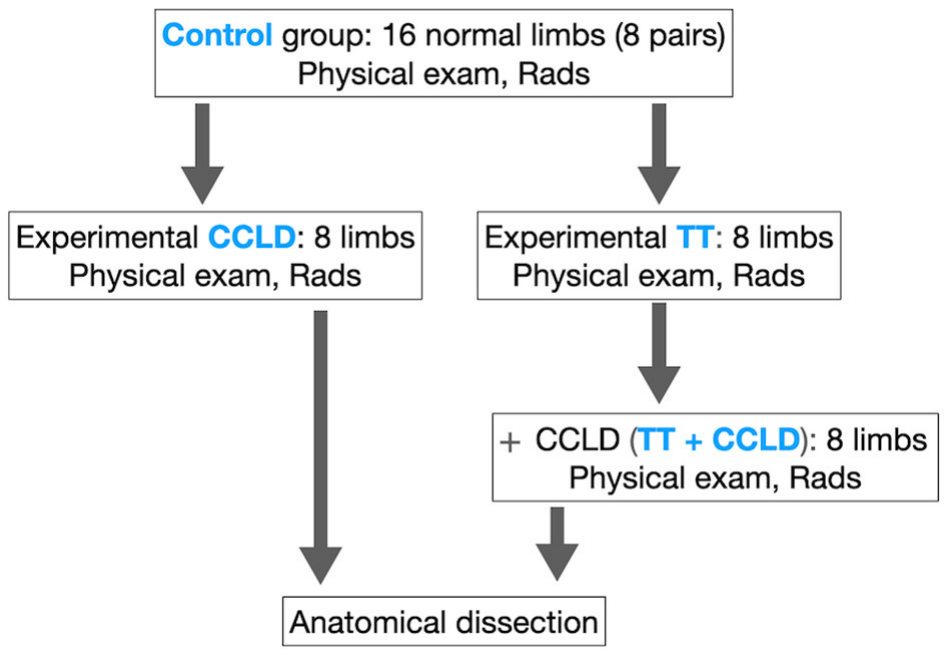
Study design. TT, induced tibial torsion; CCLD, induced cranial cruciate ligament deficiency; Physical exam, orthopedic examination for stifle laxity and subjective assessment of limb alignment; Rads, Neutral and stress radiographs obtained with the limb in the positioning device.

Within each dog, one limb was assigned to CCLD, while the other underwent osteotomy prior to CCLD (Figure 2). Limbs were classified into 4 groups: normal, CCLD, TT, and TT + CCLD. For each group, “neutral” and “stress” caudo-cranial (CdCr) views of the tibia were obtained. Finally, the limbs were dissected to determine the anatomical tibial axis of each bone.

### Experimental models of rotational instability and tibial torsion

Within each dog, one limb was randomly assigned to CCLD (*n* = 8 limbs), as a model of internal rotational instability which was induced by transecting the CCL (18). The procedure was conducted *via* a medial mini-arthrotomy to minimize the effect on the rotational laxity of the stifle (RLS). The joint capsule, subcutaneous tissue, and skin were closed subsequently in a routine fashion prior to imaging. The contralateral limb was assigned to TT (*n* = 8) *via* transverse osteotomy of the mid tibial diaphysis of the same dog. The distal fragment was rotated internally by approximately 15° relative to the proximal segment prior to plate fixation. This degree of torsion was chosen for its clinical relevance and is consistent with a previous study (15). The degree of torsion was calculated with the chord formula and radiographic craniocaudal width of the mid-tibial diaphysis. Marks were engraved with an osteotome across the osteotomy line, as landmarks of bone alignment. K-wires placed perpendicular to the bone on both sides of the osteotomy were also used to confirm tibial torsion during surgery (19). The tibia was immobilized with a 3.5 mm dynamic compression plate and 3 cortical screws in each bone fragment. Tissues were closed routinely before radiographs. This limb was then further modified by transection of the CCLD, to simulate dogs with CCLD and TT, before repeating radiographs.

### Radiographic technique and measurements

**Figure 3:**
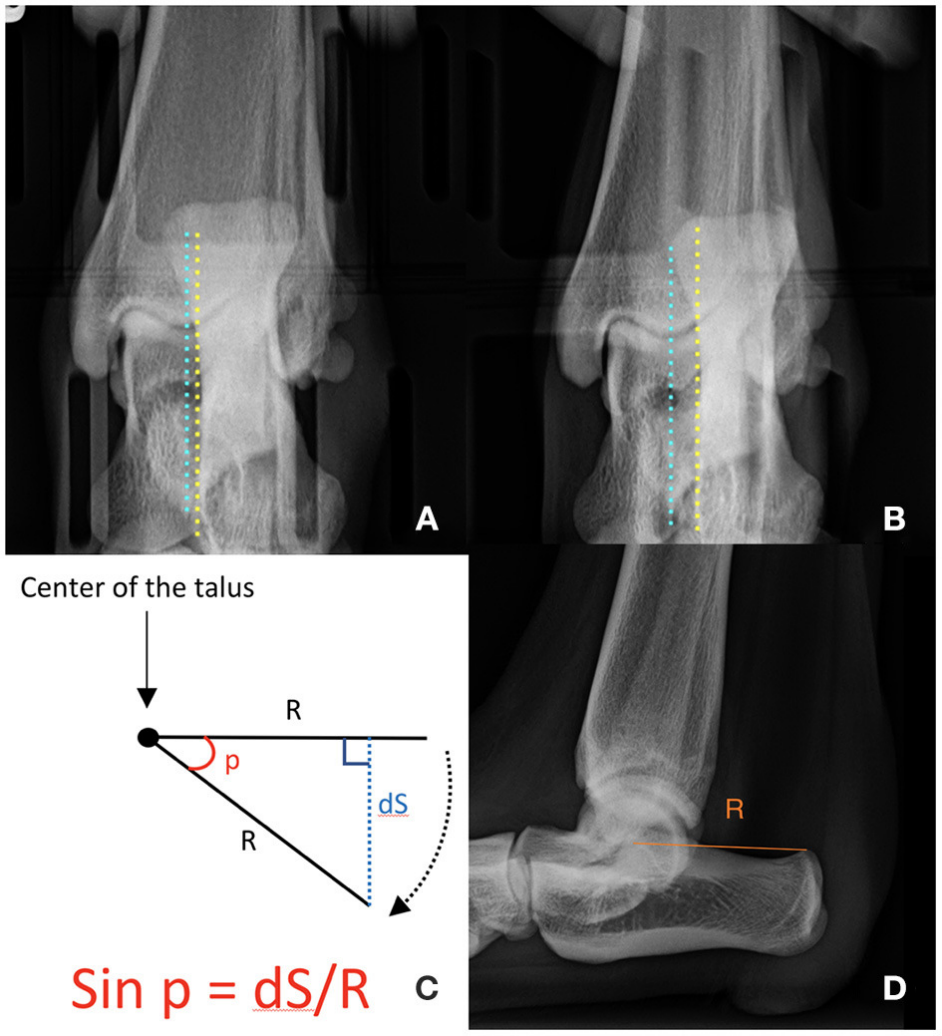
Radiographic measurements obtained on neutral and stress radiographs. Distance between the medial aspect of the calcaneus (yellow line) and the sulcus of the talus (blue line) on each neutral **(A)** and stress **(B)** view. On the neutral view, the medial aspect of the calcaneus is aligned with the sulcus of the talus (dN). On the stress view, the calcaneus is displaced laterally, increasing the distance (dS) between the two structures. The angle of rotation was calculated by using trigonometry **(C)**. The distance (R) between the center of the body of the talus and the proximo-caudal edge of the calcaneal tuber was measured on the mediolateral projection of the tibia **(D)**. We presumed that the calcaneus rotates around the center of the talus.

The radiographs were obtained using a digital radiographic system (Toshiba Rotanode?, Tokyo, Japan) with the following exposure settings: 60 kVp, 5.0 mAs, and a focal distance of 100 cm. marker was placed at the level of the limb on each radiograph to calibrate the images. Neutral and stress radiographs were obtained on intact limbs and after each modification (Figures 2, 3), with the limb in the positioning device. First, a “neutral” CdCr view of the tibia was obtained with the limb placed in the positioning device. To simulate the sternal positioning of dogs undergoing pre-TPLO radiographic examination, the positioning device was adjusted so that the tibia was parallel to the table and the stifle flexed at 135° (15). Radiographs extended from the femoral diaphysis to the metatarsus. The fabellae were symmetrically superposed and bisected by the distal femoral cortices while the distal limb was maintained in a neutral position. The “stress” view was obtained with the limb in the same positioning device while 65 N.cm of internal torque to the distal portion of the positioning device to induce internal rotation of the tibia. No tension was applied to the foot or hock. The limb was held with a bone forceps on the femoral head and adjustable rails of the device to prevent any motions when internally rotated. The thigh was maintained in neutral position, before and after medial rotation of the tibia by adjusting the rails of the positioning device. The superposition of the fabellae over the femoral cortices was symmetrical and identical on the neutral and stress views, consistent with neutral positioning of the femur.

Measurements were made on digital radiographs with an imaging software (NIH ImageJ, National Institute of Health, Bethesda, MA, USA) by two veterinarians with previous training in radiographic evaluation of canine limb conformation (JY, AM). The distance between the medial aspect of the calcaneus and the base of the sulcus of the talus was measured on calibrated neutral (dN) and stress (dS) caudocranial views of the tibia before and after each modification (Figure 3) (15, 20). Internal rotational laxity (dN–dS) was estimated based on the degree of the calcaneal displacement (mm) when torque was applied to the tibia, relative to its position under the neutral conditions. We were only able to estimate the rotational laxity of the stifle, not the rotational instability because we used the cadaveric limbs without physiological loading. The angle of rotation was estimated based on the distance (Radius) between the center of the body of the talus and the proximo-caudal edge of the calcaneal tuber on the mediolateral projection, based on the chord formula (Figure 3). Negative values were assigned to medial displacements of the calcaneus relative to the sulcus of the talus and external angles of rotation. Positive values were assigned to lateral displacements of the calcaneus relative to the sulcus of the talus and internal angles of rotation (15).

### Determination of the anatomical tibial axis

**Figure 4:**
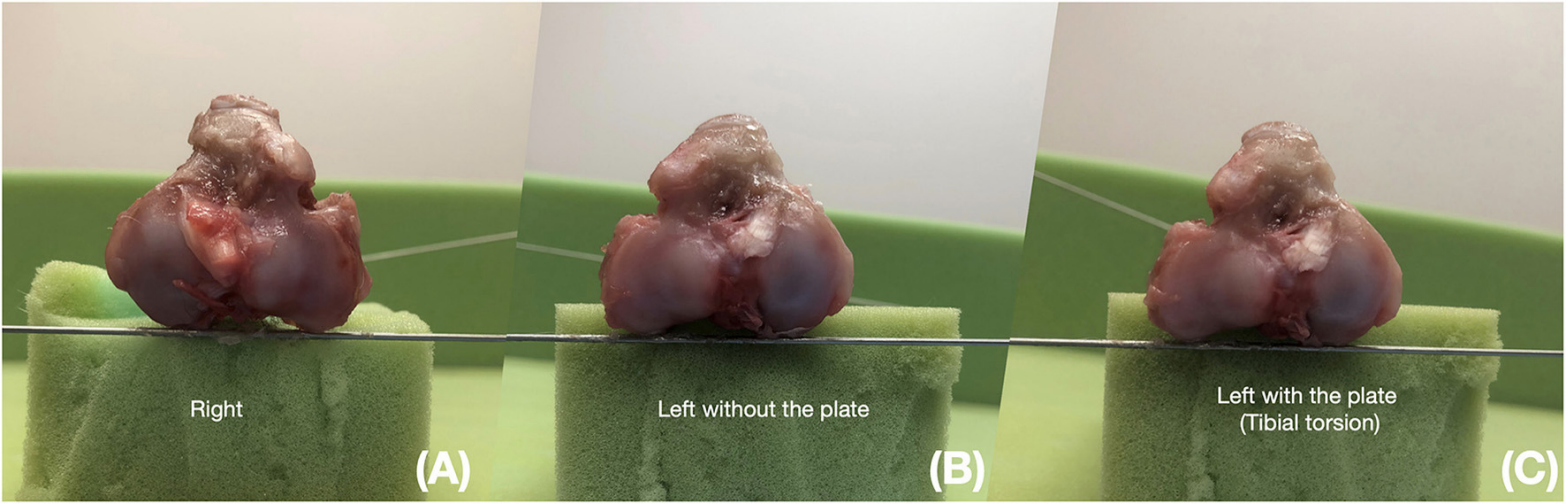
Anatomic measurements in a pair of limbs. The digital image is taken in a proximo-distal direction (axial view). K-wires are glued to the bone proximally and distally along the caudal condylar axis of the proximal portion of the tibia (CdC) and the cranial tibial axis of the distal portion of the tibia. Tibial torsion is measured as the angle between K-wires. **(A)** Right: Right tibia collected from a CCLD limb. **(B)** Left without plate: contralateral limb after removal of the dynamic compression plate and reduction of the osteotomy site. **(C)** Left with the plate: contralateral limb with the dynamic compression plate maintaining rotation at the osteotomy site.

The tibia was disarticulated and soft tissues dissected from each limb at the end of the study. Two K-wires were glued to the tibia, proximally and distally along the caudal condylar axis (CdC) of the proximal portion of the tibia and the cranial tibial axis (CnT) of the distal portion of the tibia, respectively. The tibia was positioned on a clear support and digital images were taken in a proximo-distal direction (axial view), with the camera aligned with the tibial axis (Figure 4). Plates and screws were removed, and the tibial segments were reapposed to the marks created before osteotomy to restore bone alignment. Digital images were repeated. The angle between the two K-wires was measured on all digital images to determine tibial torsion.

### Data analyses

All statistical tests were conducted using the SAS software for Windows version 9.4 (Cary, North Carolina, USA). Descriptive statistics were presented as median, first and third quartiles and range for each parameter per group. Three radiographic parameters (dN, dS-dN, and angle of rotation) were compared within dogs and between groups. A Friedman test was conducted to compare left and right intact limbs within dogs. The influence of each modification within limbs was tested by calculating the difference for each radiographic measurement between TT or CCLD limbs and the values in the corresponding intact limb. This difference was compared with median = 0 using signed rank test. The same calculation and comparison were used to compare CCLD + TT values with TT values within each dog.

Three comparisons between groups were selected, taking into consideration the clinical relevance of the study and its objectives. First, the ability of our radiographic technique to detect the presence of TT was tested by comparing all limbs with TT (combination of limbs with TT and TT + CCLD) to all limbs without TT (combination of intact and CCLD limbs). Second, the ability to detect internal rotational laxity of the stifle was tested by comparing all limbs with CCLD (combination of limbs with CCLD and TT + CCLD) to limbs without CCLD (combining intact and TT limbs). Third, the ability to distinguish the presence of TT in CCLD was tested by comparing CCLD limbs with limbs with CCLD and TT. These three comparisons were made with the Wilcoxon rank sum test. Cut-off values for the clinical use were calculated for sensitivity and specificity. Several values were produced to differentiate RLS or TT from intact limbs or to differentiate TT from RLS or to identify the presence of tibial torsion in the limbs with CCLD.

The Pearson correlation coefficient was calculated to test the correlations angles of rotation calculated on the neutral radiographs and the measured images of anatomic specimens in intact and osteotomized (TT) tibiae. All statistical tests were two-sided, and statistical significance was set at *P* < 0.05.

## Results

### Demographics

A total of 14 canine cadavers were examined and six were excluded due to abnormalities, including joint effusion (*n* = 7 limbs in 5 dogs) with or without osteoarthrosis and positive cranial drawer sign. One pair of limbs was excluded because it was tested on the initial prototype of the positioning device, which was subsequently modified. Dogs included in the study consisted of four male and four female large mixed breed dogs. Their femoral lengths ranged from 18.5 to 26.9 cm (median, 22.6 cm). Tibiae measured 18.2 to 28.2 cm in length (median, 23.2 cm) and 1.31 to 2.20 cm in width (median, 1.58 cm).

### Comparisons within dogs

**Figure 5:**
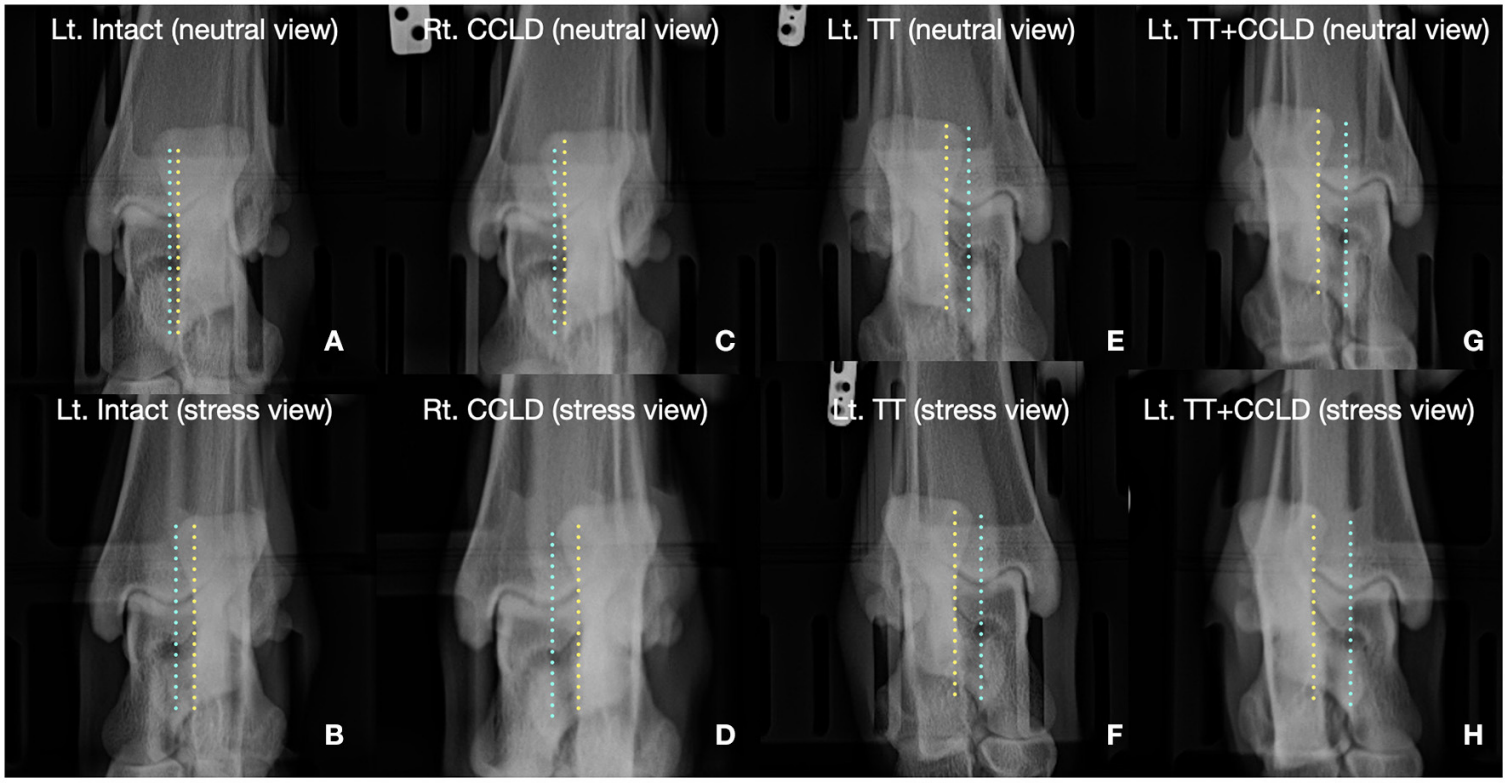
Illustrative example of radiographs obtained on a pair of limbs. **(A)** Neutral radiograph on an intact limb, **(B)** stress radiographs on the same intact limb. **(C)** Neutral radiograph on a Rt. CCLD limb. **(D)** Stress radiograph on the same Rt. CCLD limb. **(E)** Neutral radiograph on the Lt. TT limb. **(F)** Stress radiograph on the same TT limb. **(G)** Neutral radiograph on the Lt. TT + CCLD limb. **(H)** Stress radiograph on the same Lt. TT + CCLD limb. The calcaneus was laterally displaced on the stress views in all conditions, compared to the neutral views. Also, the degree of the lateral calcaneal displacement was more remarkable in limbs with CCLD than other modifications. The position of calcaneus in the neutral view was more lateral in the limbs with TT. Blue line, the sulcus of the talus; Yellow line, medial aspect of the calcaneus.

No differences were detected in dN, dS-dN, and angle of rotation between intact right and left limbs of the same pair (*p*-values > 0.05). Transection of the CCL induced an overall lateral translation of the calcaneus on neutral radiographs (dN) by a median of 0.93 mm (*p* < 0.05), although this change was not consistent (-0.7 to 2.7 mm, Table 1; Figure 5). The transverse rotational osteotomy consistently induced a lateral displacement of the calcaneus on neutral radiographs, varying between 1.3 and 8.7 mm, with a median of 3.5 mm (*p* < 0.05) (Table 1; Figure 5). Transection of the CCL did not seem to influence the positioning of the calcaneus on neutral radiographs in osteotomized specimens (TT + CCLD, *p* > 0.05). Transection of the CCL increased the magnitude of displacement of the calcaneus (dS-dN) by a median of 1.6 mm (0.3–3.1 mm, *p* < 0.05), corresponding to 3.1° of rotation (*p* < 0.05). By contrast, no difference was observed in the magnitude of displacement of the calcaneus when TT specimens were compared to intact limbs or to TT + CCLD limbs (Table 1).

**Table 1:** Comparisons between radiographic parameters after each modification within limbs.

	***P*** **-value**		
	**Intact (*****n*** = **8) vs. CCLD (*****n*** = **8)**	**Intact (*****n*** = **8) vs. TT (*****n*** = **8)**	**TT (*****n*** = **8) vs. CCLD** + **TT (*****n*** = **8)**
dN (mm)	0.0391^*^	0.0078^*^	0.1484
dN-dS (mm)	0.0022^*^	0.3984	0.6406
Angle of rotation (°)	0.0078^*^	0.7422	0.8438

dN, displacement of the calcaneus relative to the sulcus of the talus on neutral radiographs; dS, displacement of the calcaneus after application of a standardized internal torque to the distal tibia; Angle of rotation: calculated angle of rotation using dN, dS and the distance between the center of the talus and proximo-caudal edge of the calcaneal tuber; TT, tibial torsion; CCLD, induced cranial cruciate ligament deficiency.

* *P* value < 0.05 were statistically significant.

### Comparisons between groups

The calcaneus was displaced further laterally by about 3 mm on neutral radiographs (dN) when limbs with experimental TT were compared to those without TT (*p* <0.05), but the magnitude of displacement of the calcaneus (dS-dN) when internal torque was applied did not differ (Tables 2, 3). By contrast, the position of the calcaneus did not differ on neutral radiographs (dN) when limbs with experimental internal rotational laxity of the stifle were compared to those with intact CCL; however, the magnitude of lateral displacement of the calcaneus (dS-dN) tended to be greater in CCLD limbs when internal torque was applied to the distal tibia. No difference was detected in the magnitude of displacement of the calcaneus (dS-dN) in limbs with CCLD, whether or not TT was present. However, the calcaneus (dN) was located further laterally on neutral radiographs when TT was present in these CCLD limbs (*p* < 0.05).

**Table 2:** Influence of RLS and TT on radiographic measurements.

	***P*** **-value**		
	**(1) Limbs with TT (*n* = 8) vs. Limbs without TT (*n* = 16)**	**(2) Limbs with RLS (*n* = 8) vs. Limbs without RLS (*n* = 16)**	**(3) Limbs with RLS (*n* = 8) vs. Limbs with TT + RLS (*n* = 8)**
Rad DP (dN)	< 0.001^*^	0.184	0.021^*^
Rad Int R (dN-dS)	0.452	0.059	0.443
Rad Int R Cal	0.270	0.227	0.386

TT, tibial torsion; CCLD, cranial cruciate ligament deficiency; Rad DP (dN), radiographic displacement of the calcaneus on the neutral position in relation to the sulcus of the talus; Rad Int R (dN-dS), radiographic displacement of the calcaneus when stress compared to that in the neutral view; Rad Int R Cal, the angle calculated by trigonometry by using dN, dS and Radius (distance between the center of the talus and proximo-caudal edge of the calcaneal tuber).

* *P* value < 0.05 were statistically significant.

**Table 3:** Radiographic measurements in each group of limbs.

**Group**		**Measurements**		
		**dN (mm)**	**dN-dS (mm)**	**Angle of rotation (degree)**
Intact (*n* = 16)	Median	1.82	3.18	6.48
	First and third quartiles	(-0.73, 3.1)	(2.34, 4.02)	(4.28, 7.57)
	Range	−2.57 to 3.47	0.28 to 4.86	0.52 to 9.18
TT (*n* = 8)	Median	4.82	2.98	5.9
	First and third quartiles	(3.41, 7.33)	(1.43, 3.54)	(2.29, 7.23)
	Range	0.85 to 9.53	0.54 to 8.93	0.97 to 22.06
CCLD (*n* = 8)	Median	2.14	4.46	8.71
	First and third quartiles	(1.07, 3.32)	(3.53, 4.95)	(6.83, 9.32)
	Range	−0.47 to 3.78	2.69 to 7.54	4.98 to 14.82
CCLD + TT (*n* = 8)	Median	5.63	3.47	4.02
	First and third quartiles	(3.68, 8.56)	(1.49, 6.66)	(2.21, 9.73)
	Range	2.29 to 10.49	0.58 to 12.7	1.06 to 20.91

TT, tibial torsion; CCLD, cranial cruciate ligament deficiency; Rad DP (dN), location of the calcaneus on neutral radiographs; Rad Int R (dN-dS), location of the calcaneus under stress, compared to that in the neutral view; Rad Int R Cal, the calculated angle of rotation under stress.

A calcaneal displacement (dN) of 3.25 mm differentiated limbs with tibial torsion from intact limbs with 87.5% sensitivity and 87.5% specificity, and from those with RLS with 87.5% sensitivity and 75.0% specificity. A magnitude of calcaneal displacement (dS–dN) of 3.45 mm differentiated limbs with RLS from intact limbs with 87.5% sensitivity and 68.7% specificity. Lastly, a calcaneal displacement (dN) of 3.8 mm in limbs with CCLD differentiated the presence of tibial torsion from limbs with CCLD only with 75% sensitivity and 100% specificity.

### Correlations between radiographic and anatomical angles of tibial torsion

The median tibial torsion measured in intact tibiae was 7.75 (ranging from−4 to 15). Tibial torsion angles calculated on radiographs correlated moderately with those measured on images of bone specimens (correlation coefficient = 0.525). A medial torsion of 15.9° (11 to 31°) was measured on bone specimens assigned to TT, before plate removal. These angles correlated strongly with those calculated on the radiographs of the TT limbs (correlation coefficient = 0.722).

## Discussion

In this study, we validated the use of a stress radiographic technique to detect rotational laxity of the stifle and differentiate it from tibial torsion in an *ex-vivo* canine model. The main finding of the study was that the calcaneus was positioned further laterally on neutral radiographs (Rad DP, dN) of limbs with induced tibial torsion, including those with CCLD. This finding prompts us to accept our first hypothesis. The position of the calcaneus of the limbs with internal rotational laxity of the stifle (RLS) on neutral views did not differ from the limbs with intact CCL. Our results also provided evidence to support our second hypothesis as the magnitude of lateral displacement of the calcaneus on stress views (Rad Int R, dN-dS) was increased within limbs after transection of the CCL and tended to be greater when limbs with rotational laxity (CCLD, TT + CCLD) were compared to limbs without rotational laxity. However, the radiographic technique described here did not reliably detect rotational laxity in limbs with tibial torsion. This result could be attributed to inter-individual variations, affecting our ability to detect differences between groups. The presence of other supporting structures such as the joint capsule and lateral collateral ligament could also limit the impact of CCL transection on rotational laxity the lack of physiological loading, mobilizing active restraints, could also have contributed to our findings.

Positioning limbs for caudocranial radiographs of the tibia aims for symmetrical superposition of the fabellae and for the medial edge of the calcaneus to be aligned over the sulcus of the talus. The position of the calcaneus on neutral radiographs of intact limbs in our study varied within ~3 mm on either side of the sulcus of the talus, which is consistent with previous studies (17). Transecting the CCL increased the magnitude of displacement of the calcaneus on stress radiographs (dS-dN), compared to that obtained on the same, intact limb. This finding was expected as transecting the CCL creates internal rotational instability and laxity of the stifle, facilitating internal rotation of the tibia (3). These results validate the use of the radiographic technique described here to monitor rotational laxity of the stifle in individual dogs. Such longitudinal evaluation could allow detection of residual rotational laxity in dogs treated for medial patellar luxation or cranial cruciate ligament disease. Conversely, this process could be used to evaluate the influence of surgical techniques on RLS, by comparing pre- and postoperative measurements within treated dogs. However, the technique seems less reliable for one-time assessments of RLS. Indeed, the calcaneus was located further laterally on neutral radiographs after transection of the CCLD. This finding could reflect experimental artifacts as our device was designed to apply torque on the tibia of a single limb, without interference with the pes. In the clinical setting, traction is applied to the pes to assist in the neutral positioning of the limb. However, we previously reported similar findings in 16 Labrador Retrievers with CCLD (17). In this clinical study, the calcaneus of CCLD limbs was positioned at a mean of 2 mm (±3.1) compared to−0.7 mm (±3.1) in control limbs. Combined, these results provide evidence to suggest that a lateral displacement of the calcaneus on neutral radiographs of CCLD limbs can stem from internal stifle laxity alone, as previously suggested (15). This displacement remained within 3 mm from the sulcus of the talus on neutral radiographs of CCLD limbs in our study.

A calcaneal displacement (dN) of 3.25 mm on neutral radiographs differentiated limbs with tibial torsion from those with RLS with 87.5% sensitivity and 75.0% specificity in our study. Our findings differ from those reported by Apelt et al. (15) who concluded that standard radiographic examination did not discriminate internal TT from internal rotation of the tibia. In this study, the CCL was transected in 6 pairs of normal limbs, effectively creating rotational laxity of the stifles. Radiographs were obtained with the limbs in neutral position and after internally rotating the tibia by 15°. Tibial rotation induced a lateral displacement of the calcaneus, although tibial torsion was not changed in the limb. The tibial rotation was intentionally induced; This position differs from preoperative radiographs obtained on CCLD limbs in the clinical setting, where efforts aim at maintaining neutral limb alignment. In addition, this previous study relied on a predetermined degree of tibial rotation, with no measurement of the force required to induce such displacement. Measuring the displacement induced by a standardized torque in each specimen seems a better reflection of the laxity of the joint. Nonetheless, the clinical relevance of Apelt' study was to avoid misdiagnosing excessive tibial torsion in CCLD limbs. This question prompted the inclusion of limbs with concurrent TT and CCLD in our study. In this population, a calcaneal displacement (dN) of 3.8 mm on neutral radiographs of limbs with CCLD identified the presence induced TT with 75% sensitivity and 100% specificity. Such finding should be considered an indication for pre-operative computed tomography, to evaluate the level of torsion and plan correction. The inability to detect a greater magnitude of calcaneal displacement (dS-dN) when the CCL was transected in limbs with TT may reflect the maximum extent of calcaneal displacement that may be detected on caudocranial radiographs. Indeed, the displacement of the medial edge of the calcaneus on caudocranial radiographs only captures the frontal plane and does not take into consideration the displacement of the bone in the transverse plane. This finding suggests that the radiographic technique tested here would not help evaluate RLS in limbs with excessive tibial torsion. Additional experiments would be required to evaluate whether the detection of RLS would be improved after surgical correction of TT.

The trigonometric method used to calculate the angle of rotation of the calcaneus was selected to take into consideration the size of each dog. Indeed, the lateral displacement of the calcaneus of the caudocranial radiograph provides an absolute value preventing extrapolation of our findings to smaller dogs. By contrast, the calculated angle of rotation approximates the radius of the arc of rotation to the craniocaudal length of the calcaneus on mediolateral projections of the tibia. These angles correlated moderately with those measured on bone specimens, providing some evidence to support the application of this approach. However, this method did not allow us to detect the presence of TT or RLS in this experimental model, compared to the absolute measurements of displacement. We attribute this result to the lack of sensitivity of radiographic measurements, amplified in the formula. Indeed, this method requires three measurements obtained on three radiographs, combined into a ratio. By contrast, the displacement of the calcaneus requires only two measurements on radiographs obtained with the limb in the same positioning device.

The budget available for this study precluded inclusion of computed tomographic examination of the limbs, which may be considered a limitation since this modality has become standard of care to evaluate tibial torsion in dogs. Instead, limbs were dissected at the end of the study to determine their anatomical axis, using a method recently described to validate three-dimensional computed tomographic determination of TT in canine cadavers (21). Another limitation of the study is inherent to its *ex-vivo* nature and the origin of cadavers. The absence of historical information of these cadavers did not allow us to use gait data as a criterion for inclusion. However, the presence of a chronic gait abnormality is unlikely as normal orthopedic and radiographic examinations were required for inclusion. In addition, although we created a model of rotational instability of the stifle (RIS), we were only able to assess the rotational laxity of the stifle (RLS) because RIS cannot be assessed with cadaveric limbs. Texting laxity allows to examine only the passive envelope of motion of a joint without considering muscles and physiological loading. Thus, a future prospective study, taking into consideration the influence of active physiological loading is warranted. Finally, the positioning device was designed for large breed dogs. Further developments are needed to improve its versatility and facilitate its application in live dogs. The proximal limb may need to be held manually or with the help of positioning aids as well as sedation for live patients.

## Conclusion

In conclusion, experimental TT induced a lateral displacement of the calcaneus on neutral radiographs and transection of the CCL amplified the magnitude of this displacement when internal torque was applied to the tibia. A lateral displacement of the calcaneus exceeding 3.8 mm on neutral caudocranial radiographs of the tibia should prompt a presumptive diagnosis of tibial torsion in large breed dogs, regardless of the degree of RLS. The use of a 3D-printed rotating device for stress radiography may be considered to monitor rotational laxity of the stifle (RLS) in individual limbs, but did not reliably detect RLS, especially in limbs with induced TT.

## Data availability statement

The raw data supporting the conclusions of this article will be made available by the authors, without undue reservation.

## Ethics statement

The animal study was reviewed and approved by Institutional Animal Use and Care Committee of Western University of Health Sciences.

## Author contributions

DG designed this study, secured funding from Western University of Health Sciences, did all the surgeries, and provided in-line scientific editing of the manuscript. GW and DG designed the prototype of the 3D print rotating device subsequently adjusted with input from DG, GW, and JY. JY wrote the first draft of the manuscript and obtained all radiographic studies, read by AM and JY. FD analyzed the data for statistical significance. AM, DG, FD, and GW participated in the interpretation of data and reviewed the manuscript. All authors reviewed and endorsed the final version.
